# Aversive Self-Focus and Alcohol Consumption Behavior in Women with Sexual Identity-Uncertainty: Changes in Salivary Cortisol Stress Response Among Those who Drink-to-Cope

**DOI:** 10.1177/24705470221118308

**Published:** 2022-08-17

**Authors:** Amelia E. Talley, Breanna N. Harris, Tran H. Le, Zachary P. Hohman

**Affiliations:** 1 Department of Psychological Sciences, 6177 Texas Tech University, Lubbock, TX, USA; 2 Department of Biological Sciences, 6177 Texas Tech University, Lubbock, TX, USA

**Keywords:** sexual identity, self-uncertainty, self-focus, cortisol, alcohol use, stress, coping

## Abstract

**Background:**

Individuals who report sexual identity-uncertainty are at-risk for heavy alcohol consumption and alcohol use disorder symptomology. The current study examined the impact of states of aversive self-focus on subsequent consumption of ostensibly alcohol-containing beverages among a sample of women in early adulthood with varying levels of sexual identity-uncertainty (*N* = 75).

**Methods:**

Utilizing a 2 (*self-focus*: negative vs. neutral) × 2 (*attribution for any psychological discomfort*: external vs. none given) between-subjects design with 3 within-person assessments of salivary cortisol, both a moderation model and mixed-effects general linear model were tested.

**Results:**

States of aversive self-focus caused increases in overall consumption among women higher in sexual identity-uncertainty. Findings suggested consumption of ostensibly alcohol-containing beverages was more likely among women higher in sexual identity-uncertainty who also reported consuming beverages to cope with distress. Among women who reported higher levels of sexual identity-uncertainty and drinking-to-cope motives, salivary cortisol concentrations dampened more quickly over time, as they supposedly consumed alcohol.

**Conclusion:**

Findings demonstrate that, among women reporting sexual identity-uncertainty who are motivated to consume alcohol to forget about troubles or worries, situations which evoke states of aversive self-focus may contribute to differences in alcohol consumption in early adulthood.

## Introduction

Alcohol misuse is often observed among youth and adults who report being uncertain or unsure about their sexual orientation.^1,2^ Sexual identity-uncertainty, sometimes called *sexual orientation self-concept ambiguity*, is the extent to which aspects of one's sexual orientation are viewed as ill-defined, inconsistent, and unstable within and across time.^3^ Sexual identity-uncertainty is positively related to alcohol misuse (eg,^4,5^), even among individuals who may not self-identify as non-heterosexual. Experimental findings show that, among individuals with sexual identity-uncertainty, situations that explicitly focus on their sexuality contribute to bias in approaching alcohol-related cues (ie, relative to control-beverage cues), as well as greater consumption in an *ad libitum* drinking task.^6^ The current study extended prior work by testing whether: (a) general states of aversive self-focus also lead to greater consumption of an ostensibly alcohol-containing beverage among women in early adulthood who report sexual identity-uncertainty, (b) the aforementioned effect would be more robust among those motivated to drink-to-cope with psychological distress, and (c) a biophysiological stress response, namely reductions in salivary cortisol, would support the reinforcing effects of such motivated consumption.

### Sexual Identity-Uncertainty

Individuals with diverse sexual identities report various trajectories with regard to sexual identity development (eg,^7^) and the timing of developmental milestones is typically later for women, compared to men (eg,^8^). Individuals who report being “unsure” of their sexual identity are often engaged in exploring various aspects of their sexuality^4^ and may be more likely to identify as nonmonosexual (eg, bisexual, pansexual; see^9^). Individuals expressing sexual identity-uncertainty often report perceived pressure to adhere to the presumed sexual orientation binary (ie, straight vs. gay/lesbian). As a result of external and internalized pressures, sexual identity development and periods of relative sexual identity-uncertainty are theorized to be important identity-related contributors to alcohol misuse (eg,^4,10^).

Within the minority stress framework,^11^ sexual identity-uncertainty has been characterized as a proximal (ie, internal) source of minority stress that may contribute to alcohol misuse (eg,^4,10^). Proximal minority stressors are a specific, self-directed form of sexual stigma, promulgated by heterosexist norms and assumptions inherent in the larger culture, which can tax coping resources. Sexual identity-uncertainty is expected to contribute to psychological distress, a theorized mechanism (eg,^12^) that accounts, in part, for differences in alcohol use and misuse in sexual minority populations. Uncertainty-identity theory^13,14^ also provides a general motivational account of how social-identity processes can lead to risk-taking behaviors, including alcohol misuse. Specifically, when individuals feel uncertain about core aspects of their identity, such as their sexual orientation, they are believed to enter an aversive psychological state, akin to cognitive dissonance (see also,^15^), and are motivated to reduce psychological and physiological stress responses stemming from their identity-uncertainty (see^16^).

Within drinking contexts, alcohol is expected to alleviate aversive psychological states (eg,^17^) through negative reinforcement, especially as drinking behavior becomes more compulsive (eg,^18^). Specifically, when self-relevant aspects of the environment are salient, a self-evaluative process is initiated, which potentiates states of aversive self-focus when individuals are confronted with aspects of the self that are deemed unfavorable (eg,^17,19^). Alcohol consumption is theorized to inhibit states of aversive self-focus by indirectly reducing one's awareness and encoding of self-relevant “sources of tension,” (eg,^17,19^). Sources of proximal stress, including sexual identity-uncertainty, catalyzed by anti-bisexual experiences and heterosexist norms that oblige binary sexual orientations (eg,^4,15^), may contribute to psychological tension for women with sexual identity-uncertainty.

Alcohol consumption may alleviate self-relevant sources of tension brought on by increased self-focus among individuals with concealable minoritized identities (eg,^20^). Individual differences in the tendency to drink to cope with distress can exacerbate risk of alcohol misuse among adults expressing sexual identity-uncertainty (eg,^21,22^), whereby heavy drinking is used as a means to cope with feelings of psychological distress, in part, by dampening one's physiological stress response. Such accounts of motivated drinking are consistent with general models of addiction (eg,^18^) as well as those that offer intraindividual, situational accounts of risk for drinking behavior among at-risk individuals (eg,^10,12,20^).

### States of Aversive Self-Focus Cause Cortisol Stress Responses

Addiction science has sought to identify neurobiological mechanisms potentiating a person's vulnerability to alcohol misuse.^23^ The role of hormonal stress system responses (eg, hypothalamic-pituitary-adrenal [HPA] axis), in predicting consumption of and physiological responses to alcohol has received attention.^24,25^ The HPA axis regulates release of cortisol, and baseline axis activity is critical for homeostasis. This axis also aids response to and recovery from stressors^26^ and impacts health.^27^ Cortisol output, over time, has been used to examine stress responsivity in sexual minority samples in response to *acute* identity-specific and socio-evaluative stressors (eg,^28,29^). Exposure to minority-identity stressors relates to increased cortisol and other stress responses, which are linked to health disparities in at-risk sexual minority women (eg,^30^). Theoretical perspectives argue that individuals reporting identity-uncertainty who encounter states of aversive self-focus may show a greater cortisol stress response (eg,^16,31,32^), which is expected to be dampened, or reduced, following the ostensible consumption of alcohol, through negative reinforcement processes (eg,^18^).

Individuals expressing sexual identity-uncertainty are expected to encounter both general states of aversive self-focus as well as those brought on by identity-specific stressors, which tax psychological resources (eg,^30^). As argued previously, sexual identity-uncertainty functions as a potential source of proximal stress (eg,^4,11,15^). This source of internalized stress may contribute to a greater sensitivity to self-relevant sources of psychological distress for some sexual minority individuals, especially nonmonosexual women, and relate to unique physiological stress responses (eg,^33^).

### Current Study

Evidence is accumulating to support that sexual identity-uncertainty is associated with alcohol-related health disparities (eg,^1,34^), particularly among women in early adulthood. Understanding how aversive self-focus predicts drinking behavior is important because sexual identity-uncertainty is associated with alcohol-related health disparities, especially among women in early adulthood. Using a sample composed entirely of self-identified women drinkers between the ages of 21 and 35, we examined consumption behavior in a double-blind alcohol administration experiment. At the between-person level, we predicted: 1) women with higher levels of sexual identity-uncertainty would show greater consumption of an ostensibly alcohol-containing beverage after exposure to states of aversive self-focus compared to their counterparts in neutral settings, and 2) women experiencing sexual identity-uncertainty who report a higher likelihood of drinking to cope with stress would consume greater amounts in response to general states of aversive self-focus. Within-person, we expected that greater consumption among women with heightened sexual identity-uncertainty who reportedly drank to cope with stress would relate to a more robust dampening of salivary cortisol levels over time.

## Method

### Participants and Design

Participants were self-identified women who agreed to be part of an experimental study ostensibly examining “Verbal Fluency and Taste Preferences.” Participants agreed to provide saliva throughout the experimental study (*n* = 75). Women were told they would be engaging in a verbal fluency task and then rating the appeal and taste of “alcoholic beverages.” Women who reported drinking at least one alcohol-containing beverage in the past three months and who were between the ages of 21 and 35 (*M* = 23.32, *SD* = 3.14) were recruited. See Table 1 for participant enrollment and demographic information.

**Table 1.** Sample Characteristics.

Baseline characteristic	Aversive Self-focus Condition		Control Condition		Full sample	
	*N*	%	*n*	%	*n*	%
Gender						
Female	45	60	30	40	75	100.0
Race/Ethnicity						
American Indian or Alaskan Native	0	0.0	1	3.3	1	1.3
Asian American	3	6.7	2	6.7	5	6.7
Black or African American	0	0.0	3	10.0	3	4.0
Native Hawaiian or Pacific Islander	0	0.0	0	0.0	0	0
Latina or Hispanic	10	22.2	6	20.0	16	21.3
White	32	71.1	18	60.0	50	66.7
Marital status						
Single	16	35.6	5	16.7	21	28.0
Dating/partnered	21	46.7	19	63.4	40	53.3
Married	7	15.6	5	16.7	12	16.0
Divorced/widowed	1	2.2	0	0.0	1	4.0
Prefer not to answer	0	0.0	1	3.3	1	1.3
Children In Home^a^	4	8.9	1	3.3	5	6.7
Highest educational level						
Did not finish high school	1	3.3	1	3.3	2	2.7
High school/some college	9	30.0	26	57.8	35	46.6
Associate's Degree	7	23.3	2	4.4	9	12.0
University or postgraduate degree	13	43.3	16	35.6	29	38.7
Sexual Orientation						
Exclusively gay/lesbian	1	2.2	1	3.3	2	2.7
Mostly gay/lesbian	2	4.4	1	3.3	3	4.0
Bisexual, Pansexual, Demisexual	3	6.6	4	13.3	8	10.6
Mostly heterosexual	20	44.4	8	26.7	21	37.3
Exclusively heterosexual	18	40.0	16	53.3	2	45.3
Queer	1	2.2	0	0.0	1	1.3

*Note.* Participants were on average 23.29 years old (*SD* = 3.13).

Sociodemographic Characteristics of Participants (n = 75).

^a^ Reflects the number and percentage of participants answering “yes” to this question.

**Figure 1.**
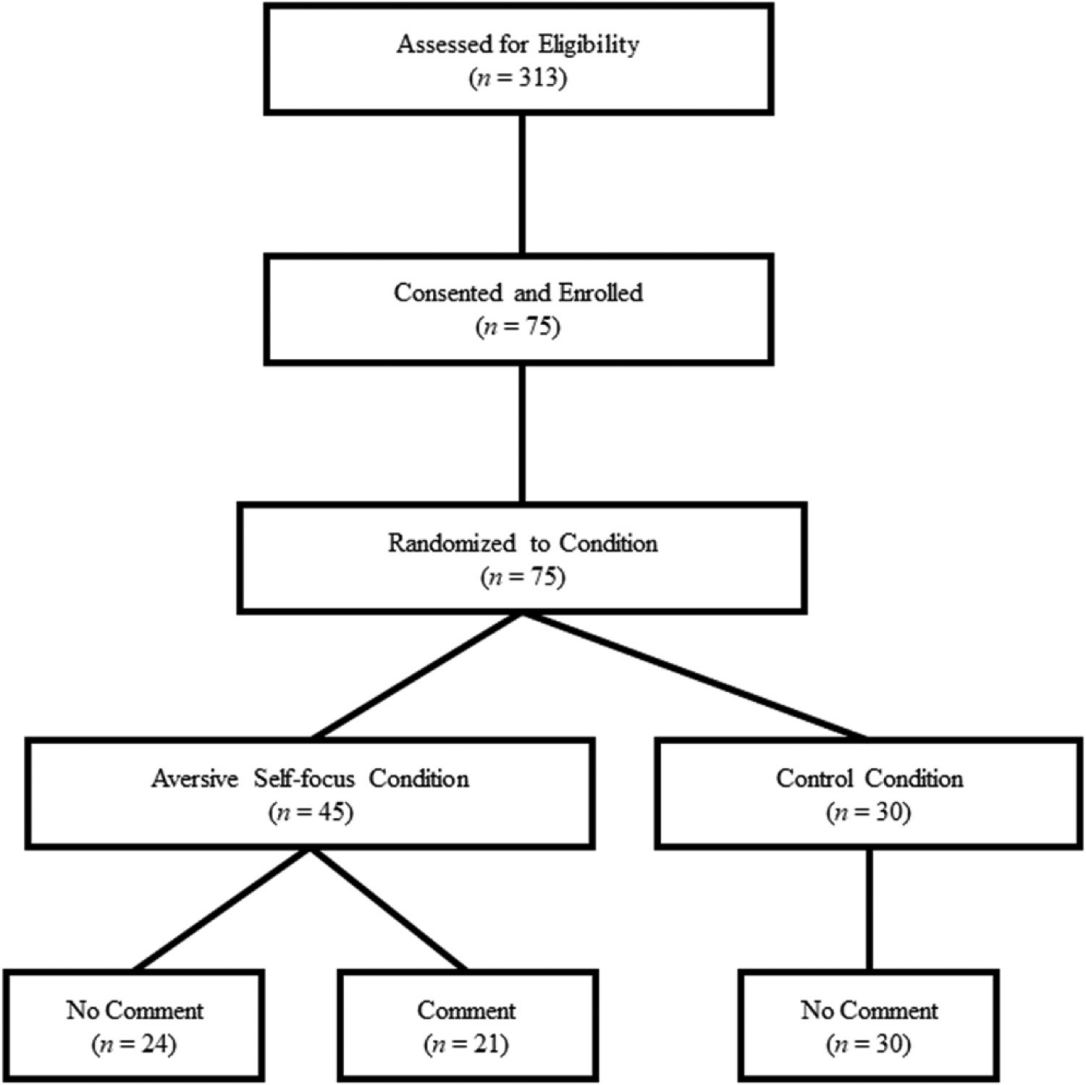
Study sample flowchart and assignment to conditions.

**Figure 2.**
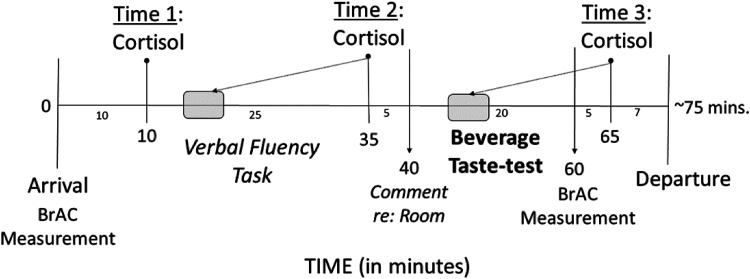
Timeline of study procedures and saliva collections. *Note*. BrAC = Breath alcohol concentration assessment. Total experimental duration was approximately 75 min per participant, salivary cortisol sample reflect baseline (Time 1), initial response to stressor task (Time 2; see corresponding shaded area), and initial response to beverage consumption (Time 3; see corresponding shaded area).

The current study was a mixed-factor design with two between-subjects factors and a repeated, within-subjects factors comprising three saliva collections throughout the course of the experiment (ie, baseline, stressor task, beverage taste test). The first between-subjects factor manipulated aversive self-focus (ie, participants were seated in front of reflective surface vs. non-reflective surface of a mirror to complete the verbal fluency task). The presence of a mirror or audience is a well-established means of evoking heightened states of self-focus in experimental studies.^35,36^ The second between-subjects factor attempted to ameliorate aversive self-focus by providing an external attribution for psychological discomfort (ie, by commenting to participants that the room where they completed the verbal fluency tasks sometimes makes people uncomfortable [*n* = 21] vs. no comment [*n* = 54]; see Figure 1). Experimental manipulation, saliva collections, and procedure timing are contained in Figure 2. All participants, who had previously identified their gender as “woman” in the pre-screening survey, were given a urine pregnancy test prior to the taste-test, regardless of their sex at birth. No other questions about gender expression or gender identity were assessed.

#### Experimental stress manipulation

All participants were asked to complete a paper-and-pencil task consisting of vocabulary and anagram questions (Supplemental Materials). To prime states of aversive self-focus during this verbal task, participants in the experimental condition (*n* = 45) were seated in front of the reflective surface of a large (3′ × 5′) mirror. Participants in the control condition were asked to complete the same solvable vocabulary and anagrams questions; however, they were seated in front of the non-reflective surface of the large mirror (*n* = 30). We did not anticipate the completion of these solvable anagrams to be particularly aversive on their own, as questions were not graded and all were solvable, corresponding to a high-school level of difficulty. In a subset of participants, a brief comment followed the verbal task and conveyed that the room often made people feel uncomfortable. We included an indicator of whether or not this comment was made as a covariate in the primary models.

### Measures and Materials

#### Beverage consumption

During the taste-test portion of the study, each participant was provided with three de-identified 12 oz. bottles of ostensibly alcohol-containing beer, labeled ‘A’, ‘B’, and ‘C’ respectively. All participants were told they were receiving alcoholic beverages and given 20 min to taste and rate the beer, with three additional beers provided upon requested. Participants answered questions regarding the taste and appeal of the drinks.^37^ The amount consumed by each participant (ml) was determined by measuring the amount of beer left in the bottles, subtracted from the initial amount given.

#### Sexual identity-uncertainty

Sexual identity-uncertainty was measured with the Sexual Orientation Self-Concept Ambiguity (SSA) scale^3^ - higher scores indicate greater acknowledgement that aspects of one's sexual orientation are uncertain. Ten items (eg, “On one day I might have one opinion of my sexual orientation and on another day I might have a different opinion.”) were rated on a Likert-type scale that ranged from 1 (‘Strongly Disagree’) to 4 (‘Strongly Agree’). Internal consistency was good in the current sample (α = .87).

#### Drinking-to-cope motivations

For each of three beers that were part of the taste test, one item asked to what extent the person could anticipate drinking the beer “to forget troubles or worries.” The three items (ie, one for each beverage) were measured with a six-point Likert-type scale (1 = Never; 6 = Almost Always). To improve reliability, the rating for Beer “B” was dropped, and the other two items were averaged to create a score reflecting the extent to which participants reported being motivated to drink to cope with distress (α = .74).

#### Salivary cortisol

Blood levels of cortisol rise within 3–10 min of stressor onset, and corresponding salivary cortisol increases shortly afterward, peaking around 20–22 min after exposure to psychological stress.^38^ Participants provided saliva samples throughout the lab session using the passive drooling method (0.5 ml per collection). The first saliva collection occurred at the start of the session, prior to any experimental manipulation and following a 10-min resting period (baseline measure, Time 1). Subsequently, a saliva collection occurred after the verbal-fluency task (ie, Time 2; 25 min following the start of the verbal fluency task), reflecting stress responses occurring during the first five minutes of the task. Finally, the last saliva collection occurred at the conclusion of the taste test (ie, Time 3; 25 min following the start of the *ad libitum* taste test), reflecting stress responses during the first five minutes of tasting of ostensibly alcohol-containing beverages.

Salivary samples were assayed in duplicate using a commercially available enzyme immunoassay kit (cortisol: 1-3002; Salimetrics, Carlsbad, CA) according to the manufacturer's instructions, as we have done previously.^39^ The standard curve ranged from 0.012 (∼92% bound) to 3.00 µg/dl (∼7% bound). Sample duplicate coefficient of variation (CV) values were all under 10%. High and low kit controls were run in each assay plate, average intra- and inter-assay CVs were 10.4% and 15.2%, respectively. Cortisol concentration was recorded as micrograms of hormone per deciliter of saliva (µg/dl); data were log transformed prior to analysis to correct positive skew.

#### Covariates

Covariates were selected because they were relevant for alternative explanations of levels of salivary cortisol (eg, time-of-day, between 10 AM and 8 PM, Median = 5 PM) or consumption behavior (eg, liking of the experimental beverages on a 9-point scale, *M* = 5.33, *SD* = 1.61; year of sample recruitment, 0 = prior to COVID-19 pandemic [2018], 1 = COVID-19 onset [2020]).

### Procedure

The pre-screening survey assessed SSA scores and sought to oversample women (a) who did not identify as *exclusively heterosexual,* (b) who acknowledged same-sex sexual attractions, or (c) who reported any history of same-sex sexual behavior, to acquire greater variation in SSA scores. All persons who completed the pre-screening survey were compensated with a $5 gift card to Amazon.com. Women who expressed any degree of SSA, as indicated by non-zero SSA scores (85% of the current sample, ^3^), or any women who reported a non-exclusively heterosexual identity (54%) or any degree of same-sex attraction (64%) or behavior (28%) in their lifetime were invited to participate.

At the start of the session, participants verified eligibility and adherence to pre-experimental protocols (ie, abstention from food, dairy, alcohol, and other drugs for 4 h before the experiment;^40^). Different experimenters were used for each task in the study such that the experimenter leading the taste-test portion of the study was blind to the participants’ assigned experimental condition.

Because we were interested in whether aversive states of self-focus precipitated the *motivation* to consume alcohol, we used non-alcoholic beer. Subjective intoxication ratings following the taste test suggested that approximately 38% of participants reported feeling “a little bit” or “somewhat” intoxicated, whereas 62% did not feel intoxicated at all. A detailed face-to-face debriefing of all hypotheses and deceptive procedures was administered. No participants indicated suspicion or guessed the study hypotheses in open-ended questions at the end of procedure, prior to debriefing (see Supplemental Materials). All relevant federal and institutional research ethical standards were met with regard to the treatment of participants.

## Results

### Descriptive Statistics

Prior to their inclusion in models, variable scores that were non-normal were transformed to improve normality (ie, alcohol consumption). Bivariate associations, as well as observed means and standard deviations, are in Table 2. One participant, who was identified as a multivariate outlier in regression models and showed residual values above 3, was excluded from analysis.

**Table 2.** Means, Standard Deviations, and Correlations among Primary Study Variables.

	SIU	Drinking-to-cope motives	Cortisol	Beverage consumption^a^
Sexual Identity-Uncertainty (SIU)	0.871			
Drinking-to-cope motives	−0.071	0.744		
Cortisol (AUC)	0.040	0.248*	—	
Beverage consumption	−0.119	0.211	−0.085	—
*M*	1.631	2.107	0.408	246.759
*SD*	0.519	0.986	0.381	196.526

*Note*. *p < .05, *n* = 74. Sexual orientation self-concept ambiguity scale scores, served as the measure of sexual identity-uncertainty. Numbers on diagonal refer to Cronbach's Alpha and were computed where appropriate for calculated scales. Untransformed variable distribution information is given. Overall output of salivary cortisol is shown.

^a^ Beverage Consumption is the average amount of beverage consumed (in ml) from each of three de-identified bottles.

### Between-Subjects Moderated Mediation Regression Analysis

#### Analytic approach

A linear regression model tested moderation hypotheses to predict overall beverage consumption. Moderation hypotheses were tested using the Hayes PROCESS Macro^41^ for SPSS, which allows for the calculation of regression effects using 10,000 bootstrapped samples to derive coefficient estimates and confidence intervals. PROCESS estimates simple slopes, for continuous scores, at the 16th, 50th, and 84th percentiles of each respective moderator, to ensure estimates are derived from observable data. Covariates included session time-of-day, presence of room comment, and liking of the taste-test beverages.

Given our expectation that women who reported higher levels of sexual identity-uncertainty and were assigned to the aversive self-focus condition would consume more beverages than in the control condition, we first tested a two-way interaction between condition and sexual identity-uncertainty (SIU) scores (see Table 3). There was initial support that states of aversive self-focus caused changes in drinking behavior and that this association was moderated by sexual identity-uncertainty. Women with higher SIU scores (ie, 84th percentile) who were assigned to the aversive self-focus condition (ie, completed the verbal task in front of the reflective surface of a large mirror) drank more in a double-blind taste test, compared to the control condition (ie, completed the verbal task in front of the non-reflective surface). No differences in conditions were shown among women who reported average or lower SIU scores (ie, 16th percentile).

**Table 3.** Between-Person Regression Model Results, Predicting Amount of Beverages Consumed (in mL).

Predictor	Coefficient	SE	t	LLCI	ULCI
Model 1: Unconditional Interaction Model					
Condition (1 = Aversive Self-Focus)	0.151	0.125	1.21	−0.100	0.402
Sexual Identity-Uncertainty (SIU)	−0.147	0.111	−1.319	−0.369	0.076
Condition*SIU	0.259	0.117	2.202*	0.024	0.493
Model 1: Conditional Effect of Condition on Consumption at Different values of Moderator					
Low SIU	−0.113	0.163	−0.692	−0.4381	0.213
Average SIU	0.086	0.125	0.687	−0.164	0.336
High SIU	0.434	0.190	2.288*	0.055	0.813
Model 2: Unconditional Interaction Model					
Condition (1 = Aversive Self-Focus)	0.200	0.129	1.55	−0.058	0.458
Sexual Identity-Uncertainty (SIU)	−0.231	0.118	−1.961	−0.467	0.004
Drinking-to-Cope Motives (DTC)	0.044	0.116	0.381	−0.188	0.277
Condition*SIU	0.312	0.128	2.444*	0.057	0.568
SIU*DTC	−0.018	0.118	−0.148	−0.254	0.219
Condition*SIU	0.092	0.113	0.815	−0.134	0.319
Condition*SIU*DTC	0.244	0.116	2.099**	0.012	0.477
Model 2: Conditional Effect of Condition*SIU on Consumption at Different values of Moderator (DTC)					
Low DTC	0.038				
Average DTC	0.286*				
High DTC	0.534**				
Model 2: Conditional Effect of Condition on Consumption at Different values of Moderators (SIU, DTC)					
Low SIU, Low DTC	0.057	0.241	0.238	−0.424	0.539
Low SIU, Average DTC	−0.102	0.162	−0.628	−0.427	0.223
Low SIU, High DTC	−0.261	0.223	−1.170	−0.708	0.185
Average SIU, Low DTC	0.087	0.171	0.505	−0.256	0.429
Average SIU, Average DTC	0.118	0.125	0.941	−0.133	0.368
Average SIU, High DTC	0.149	0.174	0.860	−0.198	0.469
High SIU, Low DTC	0.138	0.237	0.582	−0.336	0.613
High SIU, Average DTC	0.502	0.206	2.446*	0.092	0.913
High SIU, High DTC	0.867	0.297	2.920**	0.274	1.460

*Note*. *p < .05, **p < .01. Standardized coefficients are shown, *n* = 74. 10,000 bootstrapped samples contributed to 95% confidence interval estimates (ie, LLCI = Lower-limit confidence interval, ULCI = Upper-limit confidence interval), using Hayes PROCESS macro. Low values of SIU = −1.02 (ie, 16th percentile); Average values of SIU = −0.252 (ie, 50th percentile); High values of SIU = 1.09 (ie, 84th percentile). Low values of DTC = −1.123 (ie, 16th percentile); Average values of DTC = −0.109 (ie, 50th percentile); High values of DTC = 0.906 (ie, 84th percentile).

In a test of the three-way interaction hypothesis, we expected that women who reported higher levels of SIU and drinking-to-cope motives would consume the greatest amount of alcohol in the experimental condition, compared to the control condition. At average and even higher levels of drinking-to-cope (ie, 84th percentile), the respective interactions between experimental condition and SIU scores in predicting consumption were significant (Table 3). Decomposing the corresponding two-way interactions further showed that, among women with higher SIU scores who reported average and higher levels of coping motives for consumption, assignment to the experimental condition caused greater beverage consumption. Between-person results were in support of hypotheses, as they showed that women with sexual identity-uncertainty showed stronger effects of aversive self-focus on drinking behavior. Coping motives for drinking further strengthened this effect among those who reported higher levels of sexual identity uncertainty specifically.

### Within-Person Changes in Salivary Cortisol

#### Analytic approach

**Figure 3.**
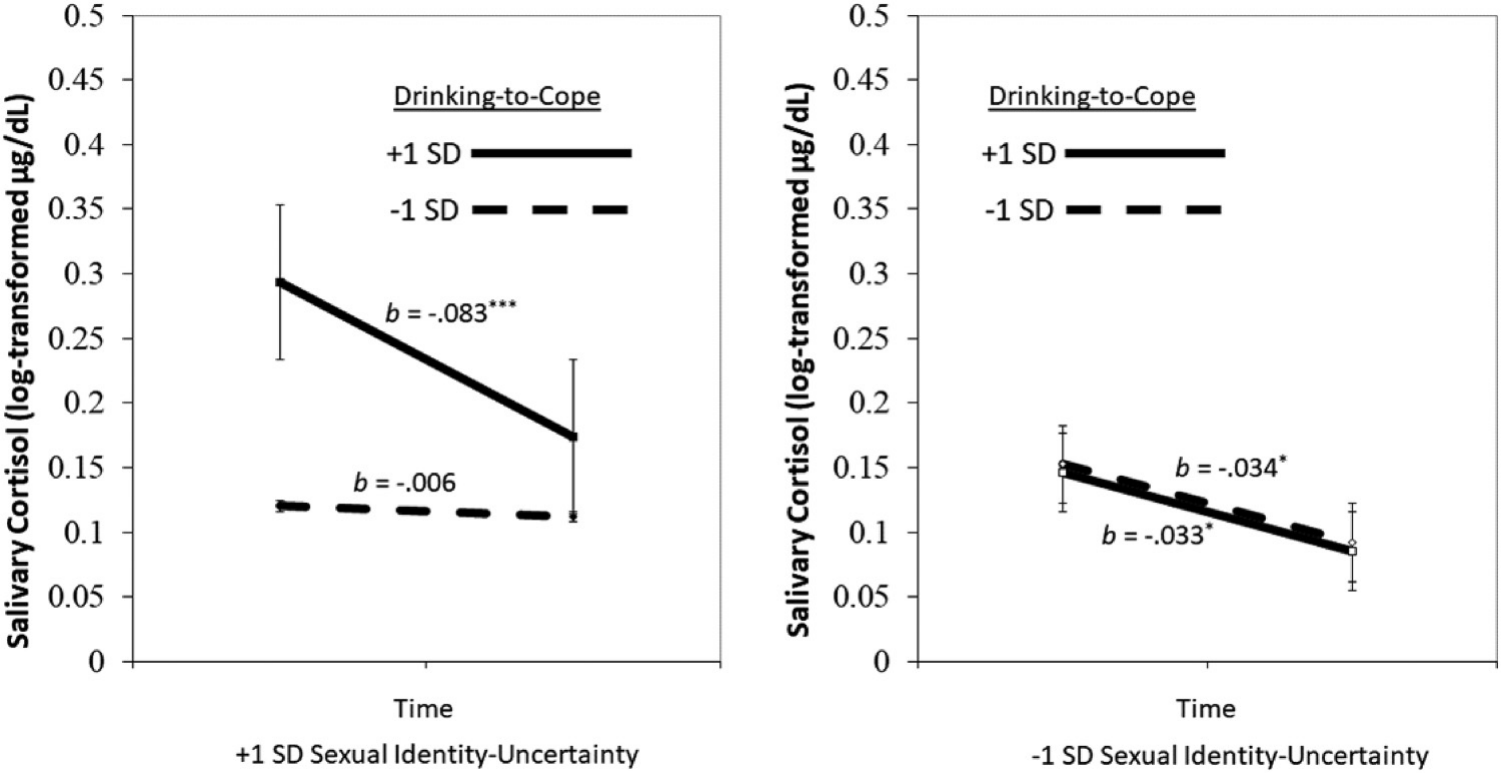
Three-way interaction between sexual identity uncertainty and drinking-to-cope predicting changes in salivary cortisol over time. *Note*. *p < .05, ***p < .001. SD = Standard deviation.

A mixed-effects general linear model (with a random intercept) was used to test hypotheses regarding within-person changes in salivary cortisol concentrations across the laboratory session among those with varying levels of sexual identity-uncertainty, see Table 4. Covariates were the same as those in the between-subjects analysis. Adjusting for consumption amount, results showed the predicted interaction between coping motives for drinking and level of sexual identity-uncertainty on changes in levels of cortisol across time, *β* = −.02, *SE* = .01, *p* = .009, 95% CI [−0.034, −0.005], see Figure 3.

**Table 4.** Mixed Effects Model, Three-way Interaction Predicting Salivary Cortisol Concentrations (log-Transformed Units).

				95% Confidence Interval	
Parameter	Effect	*SE*	*p*	Lower Bound	Upper Bound
Intercept	0.186	0.014	<.001	0.158	0.214
Time	−0.039	0.007	<.001	−0.054	−0.025
Drinking-to-Cope Motives (DTC)	0.049	0.016	.003	0.017	0.081
Sexual Identity-Uncertainty (SIU)	0.026	0.015	.076	−0.003	0.055
DTC*Time	−0.019	0.008	.020	−0.034	−0.003
DTC*SIU	0.056	0.015	<.001	0.026	0.085
Time*SIU	−0.005	0.007	.441	−0.020	0.009
DTC*Time*SIU	−0.020	0.007	.009	−0.034	−0.005

*Note*. Effect = unstandardized coefficient, estimated at median drinking levels. Sexual orientation self-concept ambiguity scale scores (SSA), served as the measure of sexual identity-uncertainty. Covariates included in the model (not shown) were liking of taste-test beverages, sample source (2018 v. 2020), time of day, self-focus condition, and mitigating-comment condition.

Changes in salivary cortisol throughout the session, in each of the randomly assigned conditions, depended on participants’ SIU scores and drinking-to-cope motives. Women who were higher in sexual identity-uncertainty (ie, SSA scores) and reported higher levels of drinking-to-cope with distress showed robust *decreases* in salivary cortisol concentrations, from the beginning of the experiment to the taste-testing session, *b* = −.083, *SE* = .014, *p *< .001, 95% CI [−0.111, −0.055], suggesting the ostensible consumption of alcohol-containing beverages dampened salivary cortisol output over time. Among those with lower levels of sexual identity-uncertainty, there were also reliable *decreases* in cortisol response over time for those lower, *b* = −.034, *SE* = .015, *p* = .023, 95% CI [−0.065, −0.005] and higher, *b* = −.033, *SE* = .016, *p* = .044, 95% CI [−0.065, −0.002], in drinking-to-cope motives, but these effects were smaller in magnitude.

## Discussion

Situations which evoke aversive states of self-focus among adults with higher levels of sexual identity-uncertainty are expected to catalyze cognitive dissonance processes (^13,15,42^) and compel drinking behavior motivated by efforts to alleviate psychological discomfort. The pattern of effects was supportive of hypotheses and consistent with theorizing that sexual identity-uncertainty may serve as an internal source of minority stress,^4^ particularly for individuals with nonmonosexual sexual identities. Women with heightened sexual identity-uncertainty are expected to regularly encounter situations characterized by states of aversive self-focus, given ubiquitous sexual stigma and discrimination from the larger society (eg,^4^). When encountering situations that evoke states of aversive self-focus and the opportunity to drink presents itself, women with heightened sexual identity-uncertainty who typically use alcohol to cope with distress may be at-risk to consume greater amounts of alcohol.

Consistent with theory (eg,^12^) and other research linking sexual identity-uncertainty to risky alcohol use,^1,4^ the current work extends prior work on approach motivations toward cues related to alcoholic, versus neutral, beverages in response to sexual identity-*specific* stressors^6^ by examining biopsychosocial stress responses to general self-relevant stressors. Prior work also suggests that elevations in distress, resulting from states of aversive self-focus among those with sexual identity-uncertainty, may be moderated by multiple factors, including identity-related factors that went unmeasured in the current report (eg, outness regarding sexual identity or sexual questioning; ^4^). Other psychosocial variables, such as alcohol expectancies (ie, individual differences in the anticipated effects of ingesting ethanol), may also alter the actual or perceived effects of stress on alcohol and relate to drinking as a means of negative reinforcement.

### Limitations & Future Directions

Findings have notable limitations. First, despite enhanced statistical power provided by within-person repeated-assessments, the current sample was somewhat small. Nevertheless, effect sizes are small-to-moderate, suggesting effects are reliable. Second, the experimental manipulation was subtle and intended to evoke general states of aversive self-focus. Prior work has supported the theorized relation between exposure to identity-*specific* minority stressors and alcohol consumption among sexual minority adults (eg,^6,43^). This is one of a few experimental studies to show that general states of aversive self-focus can also cause increased consumption among women with sexual identity-uncertainty and result in biophysiological changes in one's stress response. Results are also consistent with findings showing that distal (ie, external) LGBT-*specific* stressors positively relate to acute salivary cortisol response.^44^ Third, the current sample was composed entirely of self-identified women. Theoretical relations may be distinct based on gender or sex assigned at birth in that individual differences in stress-based hormonal systems may relate to distinct changes in salivary cortisol concentrations depending on consumption (see eg,^32^). Future research should seek to replicate these effects among individuals of different genders who are experiencing sexual identity-uncertainty. Finally, among gender minority adults, mirrors can be “triggering”^45^ and evoke distress due to gender dysphoria, or negative feelings spurned by a perceived mismatch between a person's gender identity and how others may view them.^45^ In addition, transgender and gender non-conforming adults may experience heightened body shame or otherwise respond uniquely.^46^ Although women in the current work were not asked about their gender identity, future work examining this process within samples of transgender and gender non-conforming adults may use other means of evoking self-focus, such as the presence of an audience.^36^

Findings contribute to a growing literature that examines biopsychosocial predictors of alcohol consumption and misuse among at-risk populations, including persons expressing sexual identity-uncertainty. Associations shown in the current report are expected to be even more robust when identity-specific stressors are encountered (eg,^43^) and beverages contain actual ethanol (ie, not “near beer”). Studies that have examined the impact of ethanol consumption on the HPA response, show that ethanol *decreases* the HPA axis response to stress. More data on the role of acute, voluntary alcohol consumption on the HPA axis response to proximal and distal sources of minority stress in various social settings among sexual minorities or those experiencing sexual identity-uncertainty are needed. The current work has relevance for clinical settings, as internalized sources of minority stress may be alleviated by greater societal support for exploration of nonmonosexual sexual identities.^47^ Policymakers and community stakeholders could assist in improving alcohol misuse in this population by encouraging bipositivity, refuting and dismantling stereotypes pertaining to sexual identity-uncertainty, as well as nonmonosexual identities, and enacting policies that disallow discrimination on the basis of identifying with diverse sexual identities.

## Supplemental Material

sj-docx-1-css-10.1177_24705470221118308 - Supplemental material for Aversive Self-Focus and Alcohol Consumption Behavior in Women with Sexual Identity-Uncertainty: Changes in Salivary Cortisol Stress Response Among Those who Drink-to-Cope Click here for additional data file. Supplemental material, sj-docx-1-css-10.1177_24705470221118308 for Aversive Self-Focus and Alcohol Consumption Behavior in Women with Sexual Identity-Uncertainty: Changes in Salivary Cortisol Stress Response Among Those who Drink-to-Cope by Amelia E. Talley, Breanna N. Harris, Tran H. Le and Zachary P. Hohman in Chronic Stress
