# Supplementary material for: Aversive Self-Focus and Alcohol Consumption Behavior in Women with Sexual Identity-Uncertainty: Changes in Salivary Cortisol Stress Response Among Those who Drink-to-Cope
Source: Chronic Stress (Thousand Oaks). 2022 Aug 17;6:24705470221118308. doi: 10.1177/24705470221118308 (PMC9393684; doi:10.1177/24705470221118308)
Supplement: sj-docx-1-css-10.1177_24705470221118308 - Supplemental material for Aversive Self-Focus and Alcohol Consumption Behavior in Women with Sexual Identity-Uncertainty: Changes in Salivary Cortisol Stress Response Among Those who Drink-to-Cope [file sj-docx-1-css-10.1177_24705470221118308.docx]

**Supplemental Materials: Word completion and anagram task**

Code Number:

**Directions: Please select the word that BEST COMPLETES each sentence..**

1. She dressed that way mainly to _____ a response from people because she didn't get attention since she wasn't a cheerleader.

1. LURID
2. ELICIT
3. PETULANT
4. MOOT
5. TRANSCEND

2. The fact that he used _____ to hurt my reputation instead of stating it outright makes him a despicable snake, in my humble opinion.

1. MERITORIOUS
2. INNUENDO
3. UMBRAGE
4. PROVINCIAL
5. APPROBATION

3. We formed a neighborhood _____ to fight crime, but the group eventually became a bunch of insecure fathers threatening teenagers roaming the streets at night with baseball bats.

1. ASSUAGE
2. PLENARY
3. COALITION
4. DECADENCE
5. INDUBITABLE

4. I didn't want the mortician to explain all the _____ details about the embalming process, but he felt the need to give a gruesome account of what he does.

1. PREROGATIVE
2. DECADENCE
3. HACKNEYED
4. LURID
5. PETULANT

5. I tried to _____ the pain of getting hit in the shins with a crowbar by biting my tongue, a method I learned from the Ik tribe of South Africa to alleviate pain.

1. UNCTUOUS
2. LURID
3. JADED
4. HIATUS
5. ASSUAGE

6. After the dog sniffed out the drugs, the policewoman showed her _____ by giving him a Scooby Snack.

1. PETULANT
2. PREROGATIVE
3. HACKNEYED
4. DECADENCE
5. APPROBATION

7. His parents sat him down to _____ about his plan to join the circus in an attempt to persuade him to go to college instead.

1. EXPOSTULATE
2. SIMULATE
3. APPROBATION
4. UMBRAGE
5. PETULANT

8. The pirate's parrot mimicked him _____, yes he did!... Yes he did!

1. BEREFT
2. INTIMATION
3. VERBATIM
4. GUISE
5. ADULTERATE

9. The mastermind behind the website hoped to live a(n) _____ life once the site became popular.

1. INTIMATION
2. DOUR
3. OPULENT
4. DEPLOY
5. GAPE

10. To affirm her popularity, the homecoming queen had to _____ her friends in strategic positions at the dance.

1. DEPLOY
2. DOUR
3. AUGMENT
4. VERBATIM
5. REITERATE

11. The _____ waiter was soon persuaded to give his table free appetizers.

1. PLIABLE
2. GIBE
3. DOUR
4. INTIMATION
5. GUISE

12. The _____ manager listened indifferently as the employee talked about his lateness being due to a car accident.

1. STOLID
2. GUISE
3. PLIABLE
4. GIBE
5. OPULENT

13.The _____ Bart manages never to do homework or anything else that's required of him.

1. DERELICT
2. SURVEILLANCE
3. INANE
4. INTERMITTENT
5. MERETRICIOUS

14. My _____ approach to the relationship made my date question my interest.

1. IMPETUS
2. TEPID
3. FEASIBLE
4. DUPLICITY
5. QUINTESSENCE

15. The Peace Corps. has a(n) _____ attitude towards those in need.

1. JEOPARDY
2. BENEVOLENT
3. GRIMACE
4. NOSTALGIA
5. AVERSE

16. The police had _____ evidence against the robber because many people saw him hold-up the bank.

1. LITANY
2. RECANT
3. EXACERBATE
4. IRREFUTABLE
5. SUBSTANTIVE

17. The _____ many couples share when talking and laughing with each other makes their relationship even more special.

1. RAZE
2. PORTEND
3. BADINAGE
4. FATUOUS
5. LITANY

18. The _____ with which the judge passed out the punishment was just for each crime.

1. SURVEILLANCE
2. COMPLICITY
3. INTERMITTENT
4. EQUITY
5. PERSPICACITY

19. _____ was necessary to discover who had graffitied my garage.

1. INANE
2. AGNOSTIC
3. INDICTMENT
4. SURVEILLANCE
5. PRESTIGIOUS

20. The _____ scene of the trees, flowers and hills left the woman feeling peaceful after studying the painting.

1. INDICTMENT
2. SYLVAN
3. INANE
4. ABJECT
5. INDUBITABLE

**Directions: Unscramble the letters to make a word**

1. ladut answer: _______________________

2. ssaoi answer: _______________________

3. nabci answer: _______________________

4. amcig answer: _______________________

5. vaaln answer: _______________________

6. agdeb answer: _______________________

7. ewats answer:________________________

8. alnep answer:________________________

9. wpise answer:________________________

10. diepr answer:________________________

**Supplemental Materials: Open-ended responses to Debriefing Questions** (*not a required question; original responses, including those with typos, were given by participants)

**Was there anything you did today or anything about the experimental procedure that you felt was deceptive?**

no

no

No

No

n/a

no

No

Not at all!

no

no

longer than exprected

no

nope

no

no

no

none

no

no, all information semmed necessary

yes

no

nope

no

.

no

no

no

no

no

no

no

no

no

no

no

no

no

no

n/a

no

no

no

no

I thought the time for taste evaluations was really short.

no

no

not really but there was a lot of saliva samples

**What were your expectations concerning the study?**

drinking too much alcohol

study was almost exactly what I expected.

Exactly what was expected

I didn't realize all the spitting involved

honestly thought i would be recquired to increase my BAC to above .08

no real expectations. and not much information coming in

I expected to try beer and perform some type of verbal analysis.

just as expected

i expected to taste alcohol, was not keen beer

My expectations (verbal test, taste-testing beer) were met.

i was expecting a second verbal test

i had full disclose for today's study.

i honest had no expectations for this exam/study.

I had no expectations

expectation met

i had read all the papers before hand

my expectations were that i would come in try beer see how it affected me.

vague description

I expected it to be a little more stiff, it was pretty laid back. Coming into the sutdy I knew I'd be taking a pregnancy test, breathlizer, drinking, then repeating be to check BAC.

my concern was it saying that I may drink enough to be intoxicated.

emails told a lot, yet somewhat vague

everything that happened was expected, not surprised.

I didn't know how much info on the verbal portion.

i felt well prepared

I didnt really expect anything, I just knew the basics

average expectations but i was excited

none, plenty of information, perhaps better directions

I was curious but did not know what exactly.

I figured I would have to drink the full beer so it was nice to drink how much I wanted to.

really didn't have any, I knew I would be trying different types of beer

none, just the basics from the techannounce

my expectations aligned with what happened, although I didn't know i would have my saliva tested

I thougt I would have to drink more

not much info before, I expected to taste the beers but didn't expect the puzzles

it went as I thought it would

I thought i would do a verbal test after the study, or rather the vocabulary test

it was pretty much how I expected it to be except I thought there would be other people present

everything was straight forward!

all I know was what i was told.

I only read what was on Texh Announce and in the email sent to me. The only thing I wasn't sure about was how much I would be drinking

drinking beer with a group. Where to go and when and a little of a 'what's to be expected

i didn't have any information

I didn't know much about the stud so I didn't really come in with any set expectations

That i'd come in, drink, then be quizzed.

i expected to come in and drink and have my socialization testes.

**How much information did you have coming into the study?**

read all emails sent, had tons of information and well explained

the only information I had about the study was general overall expectation of it

That the verbal test would be while drinking

just beer and verbal eval

had no clue other that it was called verbal fluency and a tast test would be involved

I had very little information abut the study

just the right amount

I was provided ample detailed information. I didn't expect the spitting, but that was optional

n/a

i had all information, was not surprised by anythign

Beside information provided in emails, I didin't know anything else

figured paperwork and beer

and it followed them well.

and coming into the study i had a good amount of info

I did feel like I had enough info.

I had enough info

But the email explained most of it

I am really interested on how these two factors of verbal fluency and alcohol can related one to another

the emails were descriptive enough

i was very imformed coming into study

I would do prior to the study and do not feel I know too much about beer than I thought I would

I had enough information to know what the study was about

no

no

na

I had sufficient information

no

no

I had no info prior to me study

i knew I would be tasting alcohol and testing "verbal fluency"

I had only the info that was provided

I knew I was drinking and that was it

I felt like I knew exactly what was going to happen coming into this

**Do you have any other comments or questions?**

yes, what is your beginning hypothesis? What do you expect to do with the information you gather?

nope

No

good luck with your research

very cool study, hope my data/information helped

nope

no

nope

This was fun!

it was very enjoyable

Will I get to know the types of beer after the study is concluded?

no

no

nope

i'd like to know the resulsts of the study when its finished and what you were hoping to find in the study

no

thank you for having me and happy to help!

nope! ;)

Thanks so much!

whould do again! or something similar

no

looking forward to seeing/hearing the debriefing! :) Thank you! This was a fun study to participate in!

none

no
